# Anæsthetics Adverse to Union by First Intention

**Published:** 1869-06

**Authors:** 


Anæsthetics Adverse to Union by First Intention.—Prof. Frank H. Hamilton, (*N. Y. Med. Record.*) expresses the opinion that union by first intention is not so apt to occur after the free use of anæsthetics; and that it "ought to be regarded therefore as one of the many causes operating to the production of suppuration and its consequences." He quotes the opinion of Velpeau, that "after the use of these agents, wounds do not heal so readily by first intention."

